# The miR-17 ∼ 92 Cluster: A Key Player in the Control of Inflammation during Rheumatoid Arthritis

**DOI:** 10.3389/fimmu.2013.00070

**Published:** 2013-03-19

**Authors:** Lucas Philippe, Ghada Alsaleh, Seiamak Bahram, Sébastien Pfeffer, Philippe Georgel

**Affiliations:** ^1^ImmunoRhumatologie Moléculaire, INSERM UMR_S 1109, Centre de Recherche d’Immunologie et d’Hématologie, Université de StrasbourgStrasbourg Cedex, France; ^2^IBMC du CNRS, UPR 9002 Architecture et Réactivité de l’ARN, Université de StrasbourgStrasbourg Cedex, France

**Keywords:** miRNA cluster, miR-17 ∼ 92, inflammation, rheumatoid arthritis, TLR, ASK1

## Abstract

MicroRNAs (miRNAs) are now recognized as essential regulators of gene expression in plants and animals. They potentially modulate the expression of multiple genes thereby enabling homeostatic settings in physiological conditions. Their role is also increasingly considered in many diseases in which deregulated epigenetic mechanisms induce aberrant gene expression. Work conducted in our laboratory has recently led to the identification of miRNAs essential for the control of inflammatory reactions that occur during rheumatoid arthritis (RA). In this review, we describe two such miRNAs, members of the miR-17 ∼ 92 cluster, which has been previously implicated in cancer. Based on our data and on predicted miRNA:mRNA interactions, we will extrapolate a model whereby the miR-17 ∼ 92 cluster appears as a global regulator of the Apoptosis Signal-Regulating Kinase 1 signalosome, a central actor in the inflammatory pathways activated during RA. We will also discuss the potential therapeutic outcomes emerging from this model.

## Introduction

MicroRNAs (miRNAs) are small (∼22 nt long) non-coding RNAs that appear central to the adjustment of gene expression in a large number of multicellular as well as a few unicellular organisms (Krol et al., 2010). Upon binding of their 5′ extremity, also called the seed sequence (encompassing nucleotides 2–7 or 2–8) with a complementary site located most of the time in the 3′ untranslated region (3′UTR) of target mRNAs, miRNAs alter gene expression by various mechanisms such as translational repression or RNA degradation (Esteller, 2011; Pasquinelli, 2012). This limited base pairing interaction between miRNA and mRNA entitles that a vast number of genes may be targeted by a single miRNA, an assessment that was experimentally verified (Guo et al., 2010; Mukherji et al., 2011). Consequently, miRNA-dependent regulation has become increasingly important for most (if not all) biological mechanisms [such as development (Stefani and Slack, 2008)] and its alteration is suspected to occur in multiple pathological conditions (Mendell and Olson, 2012; O’Connell et al., 2012).

Interestingly, while multiple miRNAs are expressed individually and scattered across the entire genome, several of them are clustered and expressed as polycistronic precursors. This enables co-expression of several miRNAs at similar levels. Furthermore, it was demonstrated that clustered miRNAs are more efficient at regulating a complex pathway than separate miRNAs. Indeed, miRNAs grouped into clusters seem to act cooperatively and in a coordinated fashion to perform their regulatory functions at different levels of a gene network. The emergence of miRNA clusters is a recent event during evolution and most of them appear to be well conserved across vertebrate species. Such a high degree of conservation and stability indicates that the expression of miRNA clusters is subjected to strict regulation and that within the clusters the miRNAs are themselves functionally constrained (Sun et al., 2013). In this review, we report our recent observations of the involvement of miRNAs encoded by the cluster miR-17 ∼ 92 in the pathogenesis of Rheumatoid Arthritis (RA). Furthermore, we discuss the potential role of this miRNA cluster in the activation and regulation of the Mitogen-Activated Protein 3 Kinase (MAP3K) Apoptosis signal-regulating kinase 1 (ASK1), a central component of the inflammatory response triggered by Toll-like Receptors (TLRs) ligands in synovial fibroblasts.

## The miR-17 ∼ 92 Cluster and Cancer

Among the miRNA clusters analyzed in higher vertebrates, miR-17 ∼ 92 has been the focus of intense attention because of its role as a potential oncogene, hence its other name, OncomiR-1 (He et al., 2005). This cluster, located on human chromosome 13 (chrom. 14 in mice) encodes a polycistronic miRNA gene which is matured into six functional miRNAs, miR-17, miR-18a, miR-19a, miR-20, miR-19b, and miR-92 which are grouped in four families based on their seed sequence. The oncogenic role of the cluster miR-17 ∼ 92 was first evidenced by the observation that the corresponding locus is frequently amplified in some lymphomas and solid tumors (Mendell, 2008; Olive et al., 2010). In addition, individual miRNAs of this cluster interact with several genes and pathways involved in tumorigenesis. miRNA-dependent targeting of negative regulators of Phosphatidylinositol-3OH Kinase (PI3K) or B cell lymphoma 2 (BCL-2) family members has been described (Lujambio and Lowe, 2012). E2F transcription factors, which are critical cell cycle regulators and also control apoptosis, also appear to be regulated by miRNAs encoded by the miR-17 ∼ 92 cluster. Interestingly, these transcriptional regulators directly affect the expression of the cluster, thus establishing a negative feedback loop. Finally, c-Myc induction of the cluster contributes to its oncogenic role through the targeting of antiangiogenic factors such as thrombospondin-1 (Tsp1) or connective tissue growth factor (CTGF) (Mendell, 2008). Altogether, expression studies performed with human samples as well as investigations in several mouse models, in which the cluster miR-17 ∼ 92 was either overexpressed (Li et al., 2012) or deleted (Ventura et al., 2008), indicate that this cluster plays important roles in cancer development.

A detailed analysis of the role of this cluster in neuroblastoma cells was recently performed to understand its global role and identify miRNA target genes in a well-defined cancer model (Mestdagh et al., 2010). Using mass spectrometry to identify changes in protein contents upon tetracycline-induced miR-17 ∼ 92 over expression, perturbation in TGF-β signaling was observed at multiple levels. This study demonstrates that the miR-17 ∼ 92 cluster weakens TGF-β signaling through interaction of individual miRNAs with multiple actors of the pathway, both upstream and downstream of SMAD2 and SMAD4 transcription factors. This possibility to control several steps of a signaling cascade underscores the ability of the miR-17 ∼ 92 cluster to tightly regulate TGF-β-dependent transduction cascades. This offers a tremendous opportunity to modulate the entire TGF-β-dependant transcription program via inhibition of miR-17 ∼ 92, for instance using antagomirs.

## miRNAs and Rheumatoid Arthritis

In addition to their role in cancer, miRNAs have also been involved in the regulation of many aspects of immune responses, from the release of inflammatory mediators to antibody production or helper T cells differentiation (Tsitsiou and Lindsay, 2009; Tomankova et al., 2011). Therefore, miRNA abnormal expression was associated with several immune-related disease such as inflammatory disorders (O’Connell et al., 2012). Among those, RA is a frequent (1% of the US population is affected) chronic inflammatory autoimmune disease affecting the joints and which can lead to major distress and substantial morbidity (Duroux-Richard et al., 2012). The involvement of miRNAs in RA pathogenesis was first evidenced by the identification, in the serum of RA patients, of auto-antibodies directed against components of the P bodies, which are intracellular structures important for RNA silencing (Jakymiw et al., 2006; Bhanji et al., 2007). More recently, expression studies uncovered altered miRNA production in the serum of RA patients compared to controls. Using microarray analysis, several miRNAs exhibiting a disease-dependent modified expression pattern (either up or down modulation) were identified in the mesenchymal compartment (the synoviocyte) or in immune cells (Wittmann and Jack, 2011). Not surprisingly, miRNAs already described as important modulators of immune responses, such as miR-155 or miR-146, were identified in these studies. Importantly, a prominent role for miR-155 in experimental arthritis was observed using knockout mice, thereby confirming the major role attributed to this miRNA in RA pathogenesis (Bluml et al., 2011; Kurowska-Stolarska et al., 2011). This opened the way to novel strategies in which miRNAs can be used as biomarkers for diagnostic, prognostic, or theranostic purposes (Nakasa et al., 2011). Finally, therapeutic intervention is also considered using small interfering RNA (siRNA)-based gene silencing in order to dampen excessive inflammation or tissue damage during RA (Apparailly and Jorgensen, 2013).

## miR-19, miR-20a, and Rheumatoid Arthritis

Fibroblast-like synoviocytes (FLS) are resident cells of the synovial membrane which play an important role in RA pathogenesis (Bartok and Firestein, 2010). They express several TLRs and can therefore respond to various infectious stimuli by the expression of pro-inflammatory cytokines (Ospelt et al., 2008). Interestingly, however, these cells secrete little or no TNF-α, IL-1, or IL-18, which are major cytokines implicated in the inflammatory response in RA. After we reported the involvement of miR-346 in this inhibition (Alsaleh et al., 2009; Semaan et al., 2011), we performed a global miRNA microarray analysis of FLS activated by various TLR ligands and focused our attention on miRNAs predicted to target components of the TLR pathways. Because we noticed that members of the miR-17 ∼ 92 cluster, namely miR-19 and miR-20, were significantly down modulated in FLS stimulated with Lipopolysaccharide (LPS, the TLR4 ligand) or Bacterial Lipoproteins (BLP, a TLR2 ligand), we decided to focus on the study of the transcriptional regulation of the entire cluster. We observed that, as opposed to hematopoietic malignancies and solid tumors where expression of this cluster is frequently activated, miR-17 ∼ 92 level was down regulated in activated FLS isolated from RA patients (RAFLS) (Philippe et al., 2012b). We next demonstrated that miR-19a and b regulate TLR2 expression thereby reducing the inflammatory response induced by BLP in FLS and which is characterized by the secretion of IL-6 and Matrix metalloproteinases (MMP-3).

More recently, we reported that miR-20, another member of the miR-17 ∼ 92 cluster, also exhibits anti-inflammatory properties. Upon targeting Ask1 mRNA, miR-20 effectively controls inflammatory cytokine production by FLS in response to stimulation by the TLR4 ligand, LPS (Philippe et al., 2012a). The resulting miR-20-dependent ASK1 repression also decreases the capacities of FLS to secrete Il-6 or MMP-3. Previous report indicated that the production of LPS-induced inflammatory cytokines, such as TNF-α, IL-6, and IL-1β, is attenuated in ASK1-deficient splenocytes and dendritic cells and that ASK1-deficient mice are resistant to LPS-induced septic shock (Matsuzawa et al., 2005). Recently, a critical role for ASK1 in the development of RA and TNF-α-induced production of inflammatory mediators in the joint was reported using different animal models (Terauchi et al., 2005; Mnich et al., 2010). This raises the possibility that ASK1 might represent a novel therapeutic target in RA.

## The miR-17 ∼ 92 Cluster, a Global Regulator of the ASK1 Signalosome?

MAP Kinases are essential components of signaling pathways involved in many cellular processes controlling cell fate: survival, proliferation, or apoptosis (Plotnikov et al., 2011). Their activation leads to the production of pro-inflammatory molecules which, when produced in excess or in an uncontrolled fashion, can cause auto inflammatory diseases such as RA. ASK1, a member of this family of kinases, is activated in response to various stress signals, including LPS or TNF-α, which generate the production of Reactive Oxygen Species (ROS) by an NADPH Oxydase 4 (Nox4)-dependent mechanism (Park et al., 2004; Zhao et al., 2007; Ngkelo et al., 2012). Upon ROS production in the cytoplasm, ASK1 unbinds from thioredoxin (Trx), which enables its autophosphorylation and subsequent activation following TRAF2 and TRAF6 binding. The complex formed by ASK1 and Trx is designated as the signalosome (Fujino et al., 2007).

Downstream of ASK1, additional MAP kinases, such as MAP3K1 (also named MEKK1), are activated and the signals converge on c-Jun N-terminal kinases (JNK) and p38 MAPKs (Soga et al., 2012).

Furthermore, regulation of ASK1 expression is performed at different levels. At the transcriptional level *Ask1* is a target of the E2F family of transcription factors. ASK1 expression is also regulated at the posttranslational level through a SOCS1-dependent degradation process (He et al., 2006).

**Figure 1:**
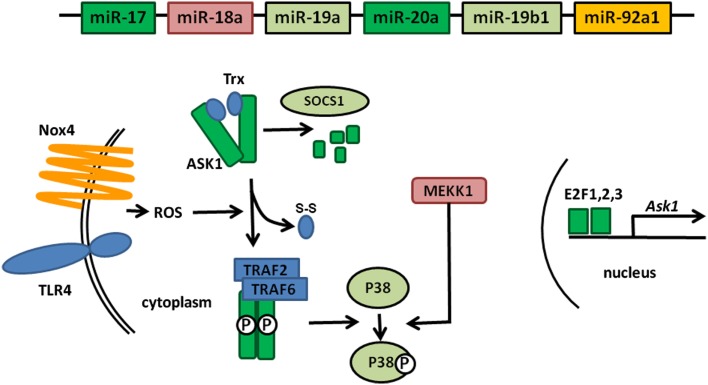
**Global targeting of the ASK1 signalosome by members of the miR-17 ∼ 92 cluster**. The individual miRNAs encoded in the cluster are represented by boxes whose color corresponds to the components of the ASK1 signalosome that they are predicted to target. Proteins shown in blue (TLR4, Trx, TRAF2, and TRAF6) do not contain miRNA-binding sites for members of the cluster miR-17 ∼ 92 in their 3′UTR.

Figure 1 gives an overview of all these partners and their roles in the regulation of ASK1. As noted above, we recently demonstrated Ask1 mRNA targeting by miR-20 (Philippe et al., 2012a). We therefore performed computational searches using TargetScan to identify miRNA-binding motifs in the 3′UTRs of additional factors involved in ASK1 activity. Interestingly, we observed that numerous components of the “extended” ASK1 signalosome (i.e., known factors involved in ASK1 activation as well as in the regulation of its expression) are potentially targeted by miRNAs encoded by the miR-17 ∼ 92 cluster. In addition to Ask1 transcripts, we noted that transcription factors of the E2F family (E2F1, 2, and 3) essential for Ask1 expression are also predicted to be targets of the miR-17/20 family. Altogether, considering that TargetScan predicts miR-92-dependent Nox4 regulation, P38 potential regulation by miR-19 and miR-18-mediated MEKK1 regulation, these observations indicate that the miR-17 ∼ 92 cluster may be a global negative regulator of ASK1 activity. Surprisingly, we observed that SOCS1, which participates in ASK1 degradation, can as well be targeted by miR-19, an effect opposite to those previously predicted since miR-19: SOCS1 transcripts interaction would lead to increased ASK1 levels. However, this might also reflect a balancing essential for the necessary homeostasis during MAP kinase activation.

As schematized in Figure 1, this analysis does not pretend to be exhaustive and it is clear that many more miRNA: mRNA interactions could be predicted. Furthermore, the expression of several genes (e.g., *Nox4*, *Mekk1*) needs to be verified in FLS and the targeting of these transcripts by the presumptive interacting miRNA needs to be validated. Nevertheless, this model strengthens the current assumption indicating that targeting several component of the same pathway by different members of a miRNA cluster likely provides a more efficient regulatory mechanism.

## Concluding Remarks

Our demonstration that ASK1 regulation by miR-20a modulates p38 phosphorylation in LPS-activated RA FLS offers interesting therapeutic opportunities. In RA, p38 MAPK isoforms have been implicated in the regulation of many processes, such as production of pro-inflammatory mediators, migration, angiogenesis, osteoclast formation and differentiation, and IL-17 signaling (Schieven, 2009). p38 involvement was demonstrated in several animal models of RA and this has led to the development of inhibitory molecules, which have recently been reported to be efficient in several arthritis models, although their clinical application remains to be cautiously evaluated because of the significant side effects of these compounds (Cohen and Fleischmann, 2010; Bonilla-Hernan et al., 2011). Targeting upstream signaling mediators, such as ASK1 might minimize undesirable off-target effects. In addition, our model predicting that several components involved in ASK1 functions are targeted by different members of the cluster miR-17 ∼ 92 creates multiple possibilities to modulate this pathway using miRNA agonists or antagonists. Several approaches have recently been suggested to deliver miRNA modulators (either mimics or antagomiRs) but in most cases, this appears a difficult task because access to the precise cellular target is highly challenging. In the case of RA, such pharmacological approach aiming at the modulation of miRNAs activity in the synovial compartment to affect inflammatory responses of FLS and macrophages may be more realistic.

## Conflict of Interest Statement

The authors declare that the research was conducted in the absence of any commercial or financial relationships that could be construed as a potential conflict of interest.
